# Metformin improves polycystic ovary syndrome in mice by inhibiting ovarian ferroptosis

**DOI:** 10.3389/fendo.2023.1070264

**Published:** 2023-01-23

**Authors:** Qingjie Peng, Xiaojiang Chen, Xiaoxia Liang, Jiahui Ouyang, Qiangqiang Wang, Shuai Ren, Haibo Xie, Chunhong Wang, Yaqun Sun, Xin Wu, Hetao Liu, Changchun Hei, Miao Sun, Qing Chang, Xinrui Liu, Guangyong Li, Rui He

**Affiliations:** ^1^ Key Laboratory of Fertility Preservation and Maintenance of Ministry of Education, School of Basic Medical Sciences, Ningxia Medical University, Yinchuan, China; ^2^ The General Hospital of Ningxia Medical University, Ningxia Medical University, Yinchuan, China

**Keywords:** metformin, polycystic ovary syndrome, ferroptosis, SIRT3, GPX4, AMPK/mTOR

## Abstract

**Background and objective:**

PCOS is a common metabolic disorder in women of reproductive age, which pathogenesis is very complex. The role of ferroptosis in PCOS is a novel finding, and the mechanistic studies are not clear. Metformin is a commonly used drug of PCOS but few studies on whether metformin can improve the follicle development and ovarian function in PCOS. We aims to use PCOS mouse model to study the effect of metformin on PCOS based on the ovarian function and explored the regulation of metformin in PCOS mice by intervening in ferroptosis pathway.

**Materials and methods:**

C57 BL/6J female mice aged 4-5 weeks were purchased and gavaged with letrozole (1 mg/kg/day) combined with high-fat diet for 21days to establish PCOS model, and control group was set up. After modeling, the mice were divided into PCOS model group and metformin treatment group (Met) (n=6).The Met group were gavaged metformin (200 mg/kg/day) for 28 days. The body weight, estrous cycle, glucose tolerance test (OGTT)and insulin resistance test (ITT) were monitored. Then, The mice were euthanized to collect serum and ovaries. Elisa was used to detect changes in related serum hormones (E2, LH, FSH, TP). Ovaries used for molecular biology experiments to detect changes in GPX4, SIRT3, AMPK/p-AMPK, and mTOR/p-mTOR by Western blot and qPCR.

**Results:**

Compared with the model group mice, body weight was significantly reduced, and their estrous cycle was restored in Met group. The results of OGTT and ITT showed an improvment of glucose tolerance and insulin resistance. Morphological results showed that after metformin treatment, polycystic lesions in ovaries were reduced, the ovarian function was restored, and the expressions of SIRT3 and GPX4 were elevated. WB results demonstrated that the expressions of p-mTOR and p-AMPK in ovaries were significantly reduced in Model group, but reversed in MET group.

**Conclusion:**

Our study confirmed metformin could not only improve body weight and metabolism disorders, but also improve ovarian dysfunction in PCOS mice.In addition, we explored metformin could regulate ferroptosis to improve PCOS *via* the SIRT3/AMPK/mTOR pathway. Our study complements the mechanisms by which metformin improves PCOS.

## 1 Introduction

Polycystic ovary syndrome (PCOS) is a common metabolic disorder, affecting 6%-10% of women of gestational age worldwide (1). About 50%-70% of PCOS patients also exhibit insulin resistance and hyperandrogenemia (2). Metformin, a classic insulin sensitizer, has been recommended by the American Diabetes Association (ADA) as a first-line medicine for the treatment of type 2 diabetes (3) and PCOS (4). Metformin can improve insulin resistance and obesity,hyperandrogenism, lower serum testosterone levels, and significantly affect PCOS (5–8). However, the underlying mechanism is complex.

Ferroptosis is a new type of iron-dependent programmed cell death (9), which has been proven to be the center of various metabolic pathways and to play a role in the occurrence and development of numerous diseases, including cancer, neurodegenerative diseases and inflammation (10–13). More and more studies indicate that ferroptosis is involved in the occurrence of PCOS (14). It has been reported that SIRT3 increases autophagy and induces ferroptosis by regulating the AMPK/mTOR pathway (15). Metformin improves insulin sensitivity (5), which is related to the activation of AMP-activated protein kinase (AMPK) (16). However, whether metformin regulates ferroptosis through the SIRT3/AMPK pathway has not been reported.

In this study, a PCOS mouse model was constructed and treated with metformin to investigate the mechanisms that metformin improved ovarian function, and the role played by ferroptosis pathway during this period.And to determine it further that whether metformin could regulate ferroptosis through SIRT3/AMPK pathway. Our study could expand the research on the pathogenic mechanism of PCOS and provide new ideas and directions of treatment mechanisms for PCOS.

## 2 Materials and methods

### 2.1 Experimental animal

This animal experiment was reviewed by the Experimental Animal Welfare Ethics Committee of the Experimental Animal Center of Ningxia Medical University and complied with the Principles of Animal Protection, Animal Welfare and Ethics (IACUC). 4-5 weeks-old C57BL/6 J mice weighted 20 ± 2 g were purchased in the Laboratory Animal Center of Ningxia Medical University(10752309202000082). During the test, the mice were guaranteed to eat and drink freely. The living environment temperature was 20 ± 2 °C. The relative humidity was 60% - 80%. The experiment was conducted after one week of adaptive feeding.

### 2.2 Main reagents

High fat feed (45% fat, D12451; Research Diets, USA); Letrozole tablets (Jiangsu Hengrui Pharmaceutical Co., Ltd.); Metformin Hydrochloride Tablets (Shanghai Squibb Pharmaceutical Co., Ltd.); NDA/RNA/Protein Kit (omega); BCA protein quantitative kit (Keji Biological Company); Western blot reagent (Biyuntian Biotechnology Institute); Goat anti mouse IgG, goat anti rabbit IgG (Zhongshan Jinqiao); Fluorescent secondary antibody (Abbkine Company); β-Actin antibody (SANTA CRUZ Company); mTOR, p-mTOR, AMPK, p-AMPK (Cell Signaling); SIRT3, GPX4 (affinity company); super fast non-toxic modified pasteurization dye kit (Nanjing Jiancheng Technology Co., Ltd.).

### 2.3 Main instruments

Leica binocular upright fluorescence microscope; BX-51 upright fluorescence microscope (OLYMPUS Japan); GelDoc XR System Chemiluminescent Gel Imaging System (Bio Rad Instruments, USA).

### 2.4 Establishment and treatment of PCOS mouse model

n=6 blank control group was created. The mice in the control group were fed regular food and gavaged with normal saline daily. The mice in the control group were given a high-fat diet and 1 mg/kg of letrozole *via* gavage for 21 days to establish a PCOS model of insulin resistance and hyperandrogenism (17). The mice, after successful modeling, were randomly divided into PCOS group and metformin group (n=6). During the treatment period, the control group continued to be fed with normal feed and given normal saline; the PCOS group was fed with continuous high-fat feed and given letrozole (1 mg/kg/day) by intragastric administration, and the metformin group was given metformin by intragastric administration (200 mg/kg/day). After 30 days of treatment, the experimental mice were euthanized, serum was collected, one mouse ovary was collected for histological examination, and the other was stored in a −80°C refrigerator for molecular biology experimental research.

### 2.5 Determination of estrous cycle by vaginal smear

From the 15th day of modeling to the 24th day of treatment, the estrous cycle of mice was detected for 7 consecutive days. Test daily at the same time. Use a Pasteur dropper to absorb an appropriate amount of normal saline, rinse and absorb vaginal secretions on a glass slide, stain with an ultra-fast non-toxic modified Pasteur stain kit, observe the cell morphology under a microscope, and determine the estrous cycle.

### 2.6 Oral glucose tolerance test

On the 19th day of modeling and the 27th day of treatment, OGTTs were performed repeatedly by taking blood from the tail vein. After fasting for 14-16 h before the experiment, initial blood glucose and body weight were measured. Glucose (3 g/kg) was administered by gavage, and blood glucose was measured 15, 30, 60, and 120 min after glucose administration.

### 2.7 Insulin tolerance test

Blood was collected from the tail vein for the ITT experiment on the 21st day of modeling and the 29th day of treatment. After fasting for 4-6 h before the experiment, initial blood glucose and body weight were measured after fasting. Insulin (1 IU/kg) was administered by intraperitoneal injection, and blood glucose was measured 15, 30, 60, and 120 min after insulin administration, respectively.

### 2.8 Histomorphological detection

#### 2.8.1 Ovary dehydration embedding

Fresh ovaries were fixed in 4% paraformaldehyde for 24 hours, rinsed with running water for 1-2 hours, dehydrated by gradient alcohol, embedded in xylene, cooled naturally, sliced at 5 um, and stored at 4°C.

#### 2.8.2 H&E

Ovary slices stored at 4°C were baked at 65°C for 1-2 hours, then dehydrated by H&E staining solution, washed with running water to remove the ethanol solution, dipped in hematoxylin for 4 minutes (depending on the quality of the hematoxylin solution), rinsed with running water to an appropriate color, and differentiated with hydrochloric acid and alcohol Rinse with running water for 2 seconds, dip with eosin for 3 minutes (depending on the quality of the eosin solution), rinse with running water to an appropriate color, then pass through the H&E staining solution in sequence, and seal with neutral resin for storage. They were photographed with a Leica binocular upright fluorescence microscope.

#### 2.8.3 Immunohistochemistry

Ovary slices stored at 4°C were baked at 65°C for 1-2 hours, dehydrated by immunohistochemical ascending solution, washed twice with PBS buffer, repaired with the citric acid solution for 10 minutes, washed twice with PBS buffer after natural cooling, and washed with peroxide Enzyme blocking agent was titrated for 10 minutes, blocked with goat serum for 1 hour, diluted antibody at a ratio of 1:200, incubated overnight at 4°C, and then rewarmed at room temperature. The antibody was developed using a rabbit two-step detection kit (Zhongshan Jinqiao), and then sequentially, After immunohistochemical staining and dehydration, the slides were sealed with neutral resin and preserved. Photographed with a Leica binocular upright fluorescence microscope.

#### 2.8.4 Immunofluorescence

Ovary slices stored at 4°C were baked at 65°C for 1-2 hours, dehydrated by immunofluorescence ascending solution, washed twice with PBS buffer, repaired with the citric acid solution for 10 minutes, washed twice with PBS buffer after natural cooling, and incubated with permeabilization solution 10min, block with goat serum for 1h, dilute the antibody at a ratio of 1:200, incubate overnight at 4°C, rewarm at room temperature, wash with PBS buffer, incubated with the corresponding fluorescent secondary antibody in the dark for 1h, incubated with DAPI for 10min, and seal with anti-fluorescence quenching Seal the slides and store away from light. Photographed with a BX-51 upright fluorescence microscope (OLYMPUS Japan).

### 2.9 Western blot

Western blot detected the expression of SIRT3, GPX4, p-AMPK/AMPK, p-mTOR/mTOR. Total protein was collected from the mouse ovary using NDA/RNA/Protein Kit (omega, R6734-02), and the protein concentration was determined using BCA protein quantitative kit (Keji Biological Company, China). The same amount (30 μg) was separated by 10% SDS-PAGE gel electrophoresis and transferred to Immobilon-P Transfer Membrane (Merck Millipore, Ireland, PR05505). The membrane was blocked in 5% skimmed milk for one hour, and after the primary antibody was incubated overnight, the secondary antibody was incubated for 2h, and autoradiography was performed (18). Antibodies and ratios used are shown in **Table 1**.

**Table 1:** Antibodies of WB.

Antidoby	Dilution Rate	Company	Cat
SIRT3	1:1000	Affinity	AF5135
GPX4	1:1000	Affinity	DF6701
p-mTOR	1:1000	Cell Signaling Technology	# 2971
mTOR	1:1000	Cell Signaling Technology	# 2983
p-AMPK	1:1000	Cell Signaling Technology	# 50081
AMPK	1:1000	Cell Signaling Technology	#2532
β-actin	1:2000	abcam	sc-8432
Horseradish enzyme labeled goat anti-rabbit IgG (H+L)	1:2000	ZSGB-Bio	ZB-2306
Horseradish enzyme labeled goat anti-mouse IgG (H+L)	1:2000	ZSGB-Bio	ZB-2305

### 2.10 Gene expression analysis

For gene expression analyses, RNA was isolated from mouse ovarian by using TRIzol (Invitrogen) and reverse-transcribed into cDNAs using the First-Strand Synthesis System for RT-PCR kit (Invitrogen). SYBR Green-based quantitative RT-PCR was performed by using the Mx3000 multiplex quantitative PCR system (Stratagene). Triplicate samples were collected for each experimental condition to determine relative expression levels. Sequences for the primer pairs used in this study are listed in **Table 2**.

**Table 2:** Sequences of primers.

	Forward	Reverse
Mouse β-actin	AGCCATGTACGTAGCC ATCC	GCTGTGGTGAAGCTGTA
Mouse GPX4	CCTCCCCAGTACTGCA ACAG	GGCTGAGAATTCGTGCATGG
Mouse SIRT3	TCCCAGATGCTCTCTC AAGCCCGTC	GCCCAATGTCACTCACTACTTCCTG

### 2.11 Statistical analysis

The Prism 8.0 statistical analysis software was used to carry out statistics on the obtained data. Measurement data were presented as means ± SEM. Two-way analysis of variance (ANOVA) was used to compare the means of multiple samples. Statistical analysis of pre and postoperative data in a group was performed using a paired t test. Differences among groups were considered significant at P<0.05.

## 3 Results

### 3.1 Metformin ameliorates obesity, impaired glucose tolerance and insulin resistance in PCOS mice

**Figure 1:**
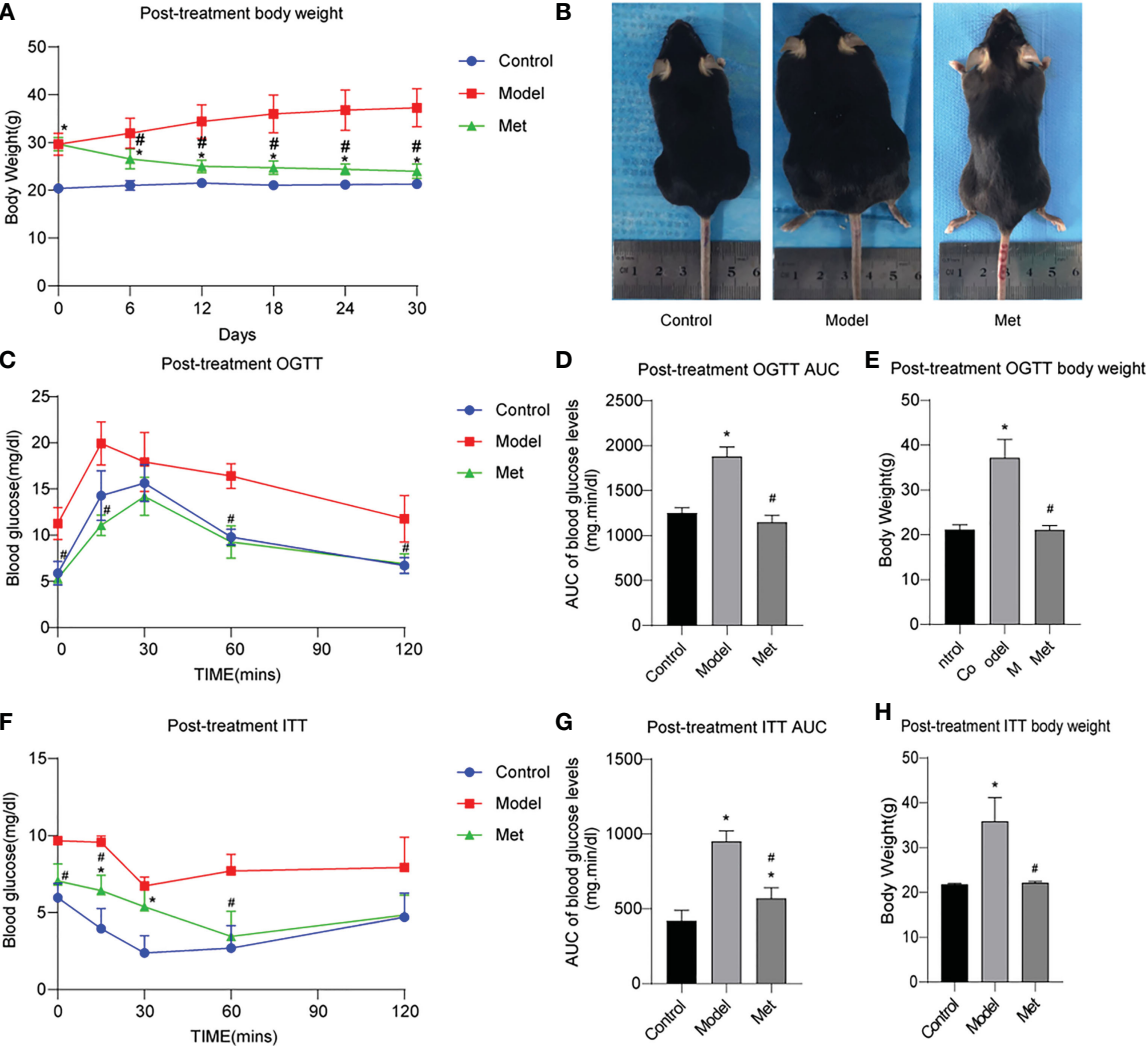
Metformin improves obesity, impaired glucose tolerance and insulin resistance in PCOS mice. **(A)** Body weight change of three groups of mice after treatment; **(B)** Body size of three groups of mice after treatment; **(C)** OGTT of three groups of mice after treatment; **(D)** Area under the OGTT curve (AUC) of the three groups of mice after treatment; **(E)** Body weight of the three groups of mice after treatment during OGTT measurement; **(F)** ITT of the three groups of mice after treatment; **(G)** Under the ITT curve of the three groups of mice after treatment Area (AUC); **(H)** The body weight of the three groups of mice at ITT was measured after treatment. *P < 0.05 vs control group; #P < 0.05 vs PCOS group. Mean = SEM, n = 6.

The body weight of the mice before and after treatment was monitored, and the body weight of the PCOS model mice was significantly higher than that of the control group (**Supplementary Figure 1A**). By comparing the area under the curve (AUC) and the body shape of the two groups, the same results were obtained (**Supplementary Figures 1B, C**); After being given metformin treatment, the body weight of the mice decreased significantly (**Figure 1A**), and the same result was obtained by comparing the body size of the mice (**Figure 1B**). The glucose tolerance of the mice in the PCOS group was significantly impaired, and the insulin resistance increased (**Supplementary Figures 1D–I**); After metformin treatment, the abnormal glucose tolerance and insulin resistance of PCOS mice were significantly improved, and the glucose tolerance had become normal (**Figures 1C–H**).

### 3.2 Metformin improves ovarian function and morphology in PCOS mice

**Figure 2:**
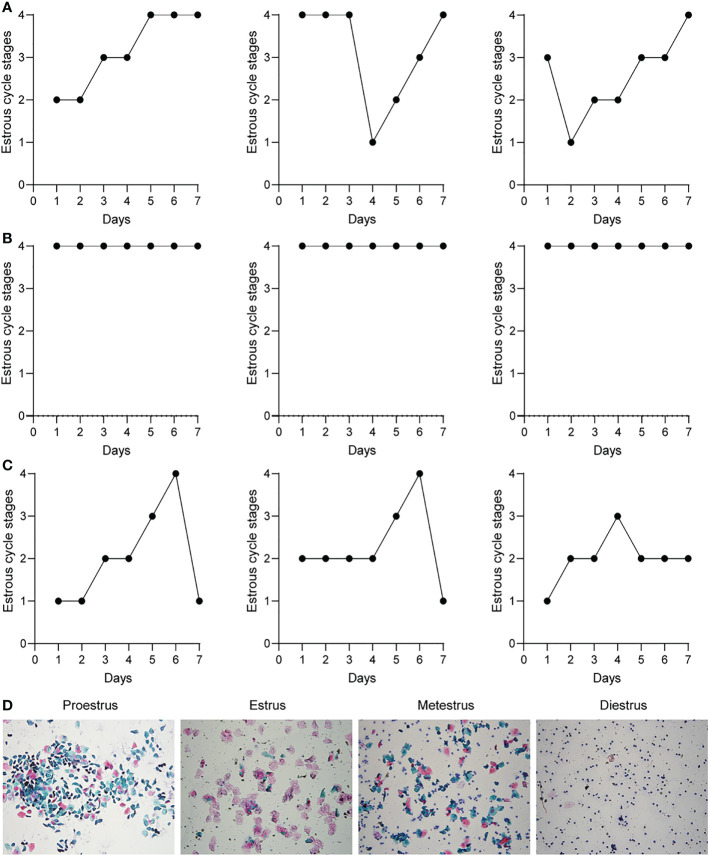
Metformin improves ovarian function in PCOS mice. **(A)** The estrous cycle of three representative mice in the normal group after treatment for 7 consecutive days; **(B)** The estrous cycle of three representative mice in the PCOS group after treatment for 7 consecutive days; **(C)** The three representative mice in the Met group after treatment The estrous cycle of 7 consecutive days; **(D)** Pap staining of mouse estrous cycle smears. The ordinate is as follows: 1. Pre-estrus; 2. Estrus; 3. Estrus and 4. Interestrus.

After the female mouse is sexually mature, there will be an apparent periodic estrus process under the action of hormones. The estrous cycle reflects the ovulation and endocrine functions of the female mouse ovary. The estrous cycle was detected seven consecutive days before treatment, and the estrous cycle of the mice in the model group was stagnant in the interphase (**Supplementary Figure 2**). The estrous cycle of the mice was detected for seven consecutive days before sampling. It was found that the mice in the normal group (**Figure 2A**) and the treatment group (**Figure 2C**) had pre-estrus, estrus, post-estrus, and interestrus, and 4-5 days were a cycle; the PCOS (**Figure 2B**) model mice were in the interestrus phase for seven consecutive days. The smears of vaginal secretions of mice were stained with a modified Pap test kit (**Figure 2D**). A large number of scattered leukocytes and a small amount of mucus were found in the smears of the PCOS group; a large amount of blue was seen in the smears of the normal group and the treatment group in the proestrus stage. In addition to nucleated epithelial cells and a small number of pink keratinocytes, a large number of pink keratinocytes can be seen in smears in estrus and nucleated epithelial cells. Keratinocytes, and leukocytes can be seen in smears in late estrus, and the number is close to 1:1:1.

**Figure 3:**
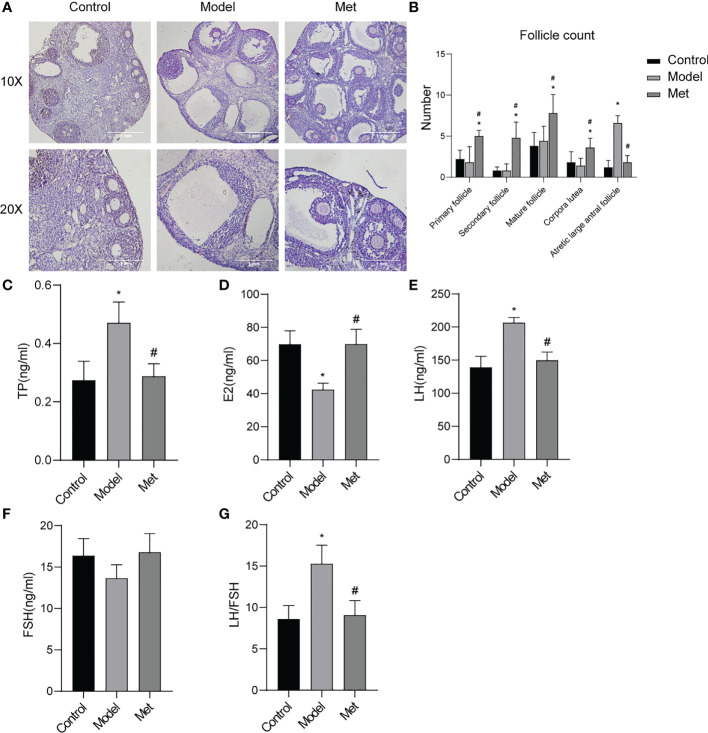
Metformin improved the ovarian morphology and function of PCOS mice. **(A)** H & E staining of mouse ovary; **(B)** Mouse ovarian follicle count; **(C)** Serum testosterone (TP) level in mice; **(D)** Serum estrogen (E2) level in mice; **(E)** Serum luteinizing hormone (LH) level in mice; **(F)** Serum follicle stimulating hormone (FSH) level in mice; **(G)** LH/FSH. Mean ± SEM, n = 6. *P < 0.05 vs control group; #P < 0.05 vs PCOS group.

The morphology of mouse ovaries was observed by H&E results showed (**Figure 3A**) follicles of all levels (primordial follicles, primary follicles, secondary follicles, antral follicles) and corpus luteum were seen in the mice in the normal group, and the structures of follicles and corpus luteum at all levels were complete, and the shapes of various cells were clearly distinguishable; Multiple large antral follicles were seen in PCOS group mice, and the granulosa cells in these large antral follicles were sparse and condensed around the follicles to form a large cavity; the ovarian structure of the mice in the treatment group is complete, the number of large antral follicles is significantly reduced, and the number of follicles at different stages of development is different. The intrafollicular granulosa cell layer was densely aggregated and thickened. The follicles were counted in the ovary (**Figure 3B**), and the results showed that the number of large antral follicles in the treatment group was significantly lower than that in the PCOS model mice, and the number of follicles at other levels was significantly higher than that in the PCOS mice. The serum sex hormone levels of the mice were detected (**Figures 3C–G**). The serum testosterone (TP) and luteinizing hormone (LH) contents of PCOS mice were significantly increased, the estrogen (E2) content was decreased, and the follicle-stimulating hormone (FSH) content was not significantly altered. After metformin treatment, TP, LH, and LH/FSH content decreased significantly, and the content of estrogen increased significantly.

### 3.3 Metformin promotes the expression of GPX4 and SIRT3

**Figure 4:**
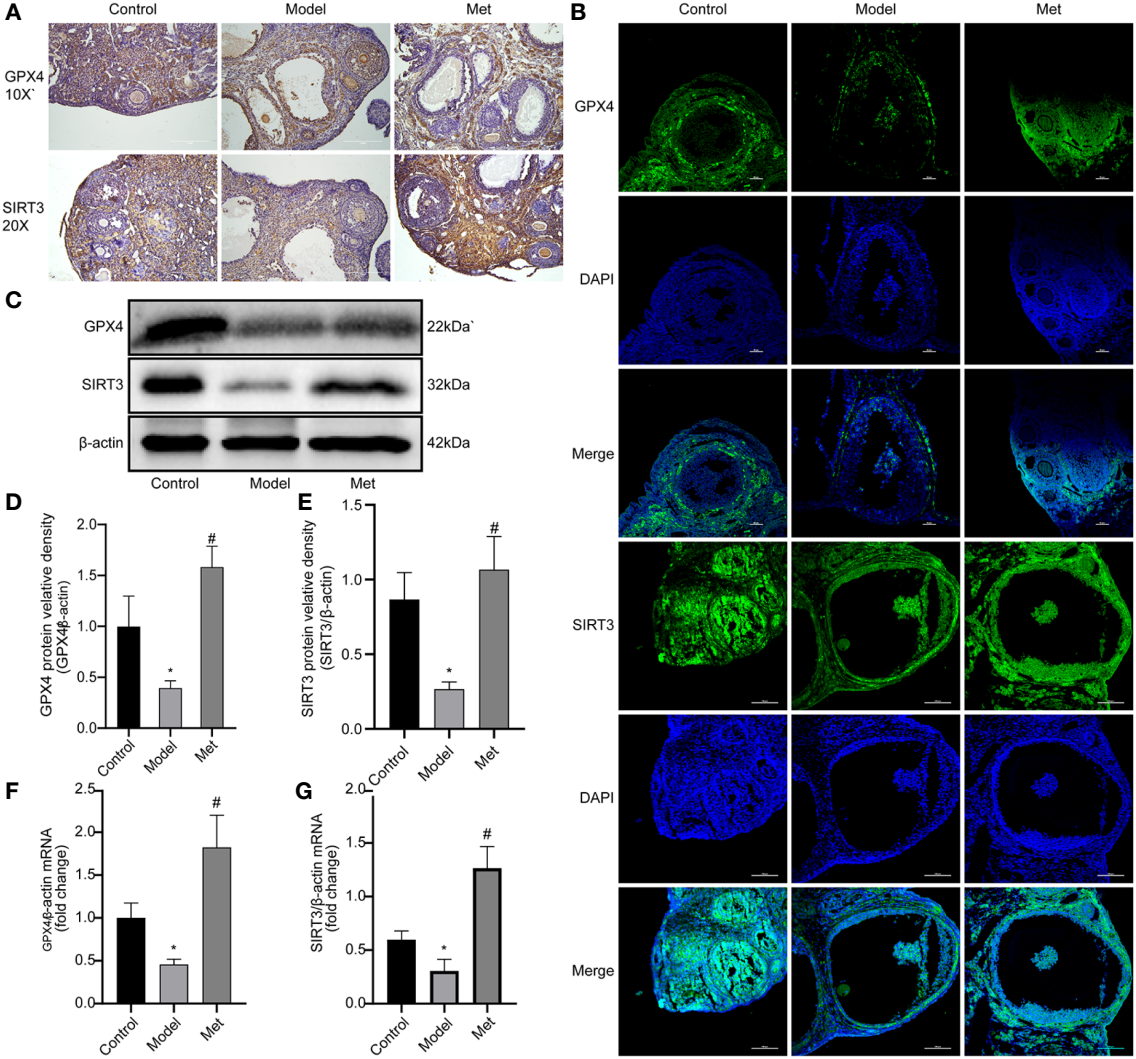
Metformin promotes the expression of GPX4 and SIRT3. **(A)** Immunohistochemistry of GPX4 and SIRT3 in mouse ovary; **(B)** Immunofluorescence of GPX4 and SIRT3 in mouse ovary; **(C)** WB of GPX4 and SIRT3 in mouse ovary; **(D)** Relative protein density of GPX4; **(E)** SIRT3 relative protein density. **(F)** qPCR of GPX4; **(G)** qPCR of SIRT3. Mean ± SEM, n = 3. *P < 0.05 vs control group; #P < 0.05 vs PCOS group.

Glutathione peroxidase 4 (GPX4) plays a key role in the occurrence of ferroptosis and is a key regulator of ferroptosis inhibition (19). Sirtuin3 (SIRT3) is a member of the sirtuin (SIRT) family member that predominantly operates in mitochondria, modulates the mitochondrial oxidative pathway, and reduce oxidative stress (20). The immunohistochemical and immunofluorescence results of GPX4 and SIRT3 showed (**Figures 4A, B**) revealed that the expression of both decreased in the ovaries of PCOS mice and increased after treatment with metformin. Mouse ovarian proteins GPX4 and SIRT3 were detected by WB and qPCR (**Figures 4C–G**), and the relative protein densities of the two were counted, and the same results were obtained. This suggested that metformin treatment could increase the expression of GPX4 and SIRT3 in the ovaries of PCOS mice.

### 3.4 Metformin activates AMPK/mTOR pathway

**Figure 5:**
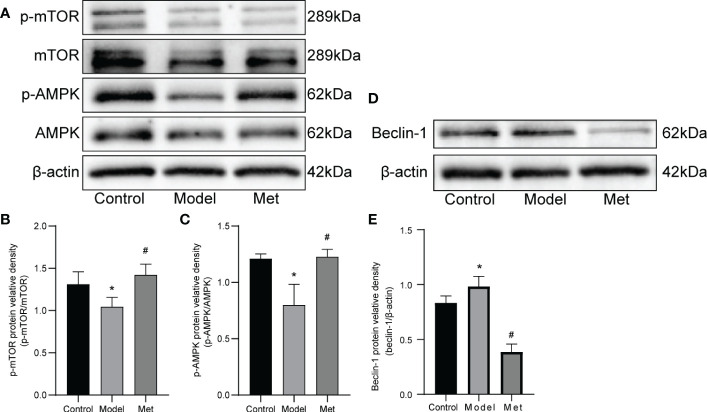
Metformin alters the expression of AMPK and mOTR in the ovaries of PCOS mice. **(A)** Western blot of mTOR, p-mTOR, AMPK, and p-AMPK. **(B)** Protein relative identity of p-mTOR. **(C)** Protein relative identity of p-AMPK. **(D)** Western blot of Beclin-1. **(E)** Protein relative identity of Beclin-1. Mean ± SEM, n=3* P < 0.05 vs control group; # P < 0.05 vs PCOS group.

WB detected mTOR, p-mTOR, AMPK, p-AMPK, and Beclin-1(**Figures 5A, D**), and the relative protein density was calculated (**Figures 5B, C, E**). The results showed that the expressions of p-mTOR and p-AMPK in the ovaries of PCOS mice were significantly decreased. After treatment with metformin, the expression of both increased. In addition, the expression of Beclin-1 increased and decreased after treatment.

## 4 Disussion

PCOS is a common metabolic disorder in women of reproductive age, characterized by insulin resistance, hyperandrogenemia, chronic inflammation, and oxidative stress, among other metabolic abnormalities (21), which also lead to ovarian ovulation disorder and abnormal ovarian function. Clinically, the primary treatments mainly aim at symptomatic relief, as the etiology and pathogenesis remain unknown (17).

Metformin (1,1-dimethylbiguanide hydrochloride) is a biguanide antidiabetic drug. It inhibits gluconeogenesis in liver, reduces glucose content, promotes the utilization of peripheral glucose and increases insulin sensitivity to decrease blood glucose levels (22, 23). In recent years, metformin has been gradually used as an insulin sensitizer in the treatment of PCOS (24). However, studies on the mechanism of metformin in improving PCOS mainly focus on the improvement of glucose and lipid metabolism disorders in PCOS patients (24–26).There are few studies on whether metformin directly improves ovarian function and regulates follicular development. The aim of this paper was first to address the effect of metformin on ovarian function and follicular development. Our study confirmed that metformin could indeed improve metabolic disorders such as body weight and blood glucose in PCOS mice. At the same time, we also found that metformin could improve the estrous cycle of mice, hormone secretion, promote follicular development and normal ovarian ovulation. However, the underlying mechanisms need to be further studied.

Ferroptosis is a mechanism of cell death mediated by iron-dependent lipid peroxidation, and oxidative stress is a fundamental manifestation of ferroptosis (12, 25). As a novel finding in the pathogenesis of PCOS, the role of the ferroptosis pathway in the development of PCOS has received increasing attention. Studies have shown that the level of ferroptosis increases in PCOS.The growth of PCOS and the occurrence of associated consequences are affected by mitochondrial damage and oxidative stress. The expression of several antioxidant enzymes, such as catalase (CAT) is decreased, and the accumulation of peroxides results in ferroptosis (24, 26). Studies demonstrate that GPX4 is a crucial regulator of iron death in both cells and mice (27). Our results showed decreased expression of GPX4 in the ovary of model mice and increased ferroptosis. In addition, metformin can protect islet cells, and after treatment with metformin, GPX4 levels increased significantly. It indicates that ferroptosis is involved in the development of PCOS, and metformin can reverse the process of ferroptosis.

Acetylation is one of the post-translational modification pathways of proteins. The acetyl group on acetyl-CoA is transferred to the lysine residue on the target protein through acetyltransferase, thereby altering the 3D conformation of the target protein and changing its biological function (28). This process can be reversed by deacetylases (29). Sirtuin3 (SIRT3) is a member of the sirtuin (SIRT) family, which mainly functions in mitochondria, mediates the mitochondrial oxidative pathway, and reduces oxidative stress (20). Studies have found that the level of protein lysine acetylation in granulosa cells of PCOS patients is increased, affecting their metabolic equilibrium (30). In our study, the expression of SIRT3 in the ovaries of PCOS model mice was decreased, and the expression of SIRT3 was restored after treatment with metformin. We delved deeper into the mechanism. SIRT3 can positively regulate the phosphorylation of AMPK (15). In the absence of SIRT3, it inhibits the AMPK/mTOR pathway, increases autophagy, and induces ferroptosis (15). Studies have shown the process that metformin directly acted PCOS (5) was related to the activation of AMP-activated protein kinase (AMPK) (16). Metformin can activate AMPK in rat granulosa cells and reduce steroid production (31). Our study found that the expressions of p-AMPK and p-mTOR were reduced in the ovary of PCOS model mice. The level of ferroptosis was inhibited by the increased expression of GPX4 and SIRT3 in the ovaries of mice following metformin treatment. In the mechanism, it was discovered that p-AMPK and p-mTOR expression increased.

In conclusion, in this study, we first addressed the effect of metformin on ovarian function and follicular development. Then, we explored the regulation of metformin in PCOS mice by intervening in ferroptosis pathway. These two points are the innovations of this paper and complement the application mechanism of metformin in the treatment of PCOS.

## Data availability statement

The original contributions presented in the study are included in the article/**Supplementary Material**. Further inquiries can be directed to the corresponding authors.

## Ethics statement

The animal study was reviewed and approved by Laboratory Animal Center of Ningxia Medical University(10752309202000082).

## Author contributions

QP, XC, XiaL, JO, QW, SR, HX, CW and YS: Data acquisition, analysis and interpretation, manuscript drafting; RH and GL: research, design and supervision, manuscript revision, funding; MS & XinL, QC, CH, XW and HL: Technical support. All authors contributed to the article and approved the submitted version.
