# Supplementary figures and images for: Metformin improves polycystic ovary syndrome in mice by inhibiting ovarian ferroptosis
Source: Front Endocrinol (Lausanne). 2023 Jan 23;14:1070264. doi: 10.3389/fendo.2023.1070264 (PMC9900736; doi:10.3389/fendo.2023.1070264)

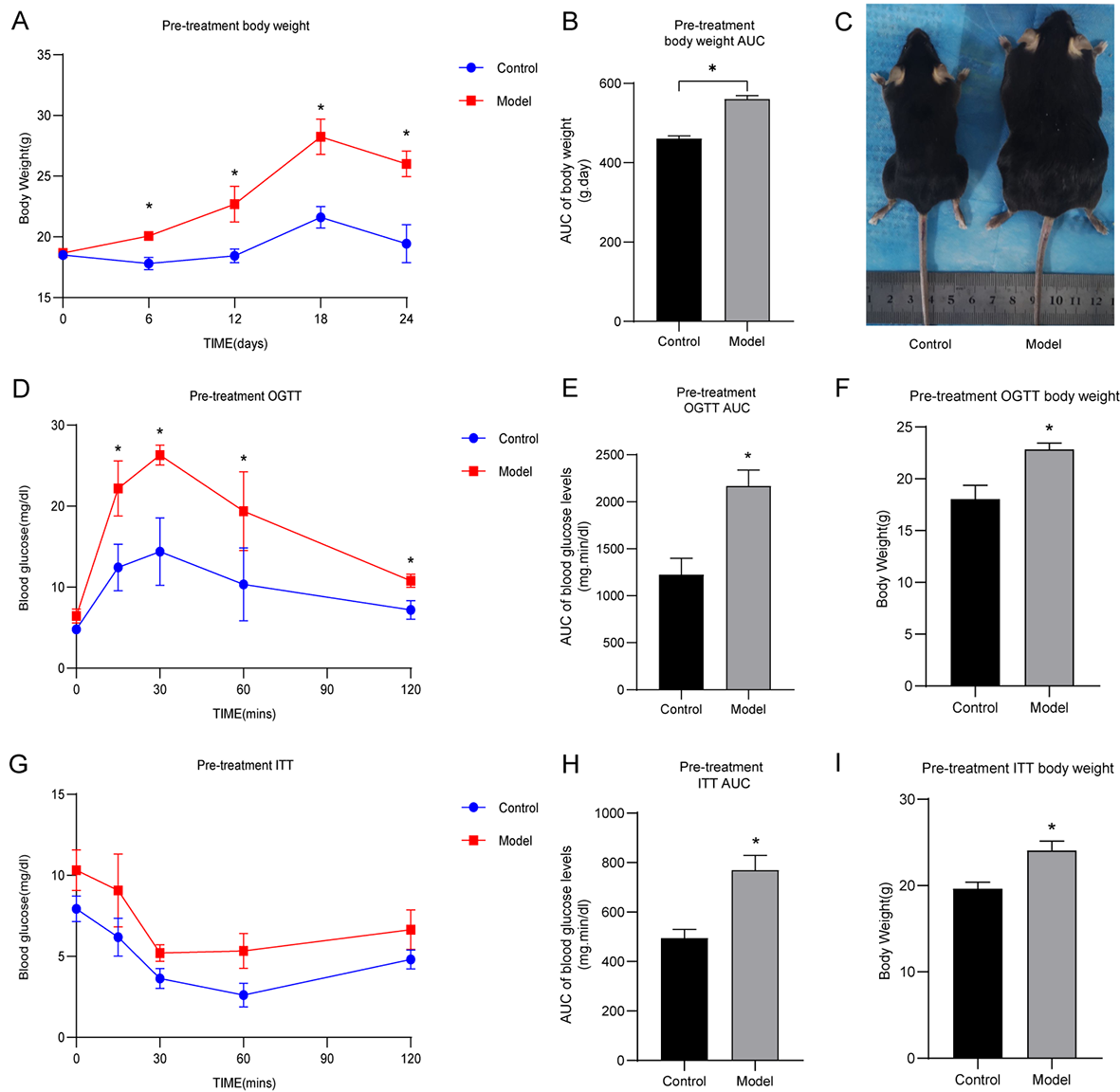

Supplement: Supplementary file 1 [file Image_1.tif]

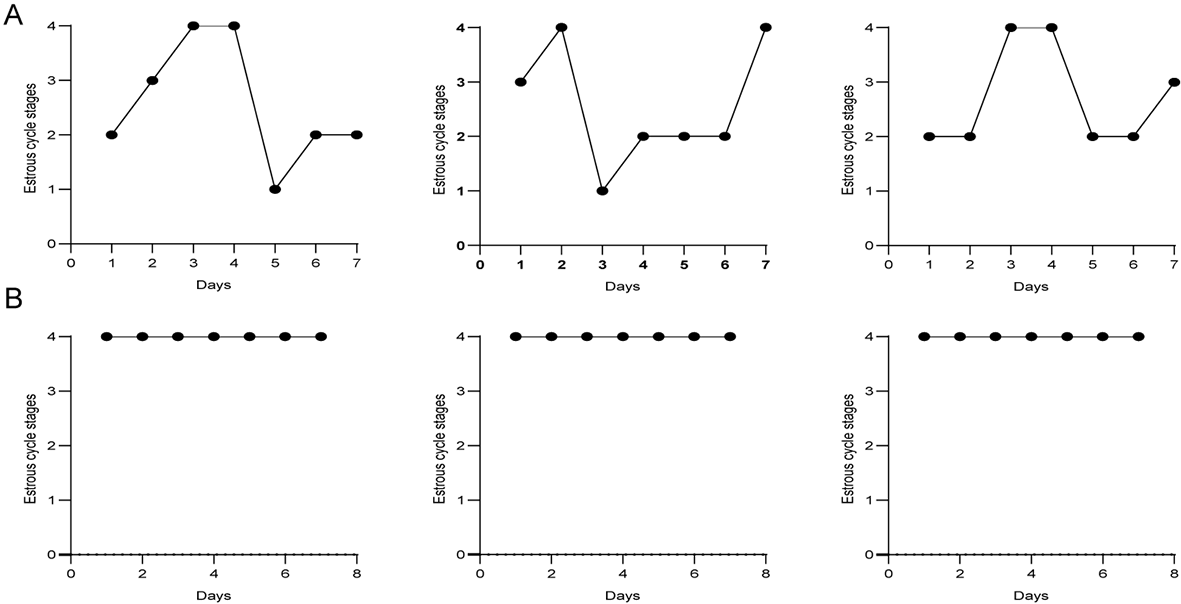

Supplement: Supplementary file 2 [file Image_2.tif]
